# Exploration of Small RNAs

**DOI:** 10.1371/journal.pgen.0040022

**Published:** 2008-01-25

**Authors:** Hideya Kawaji, Yoshihide Hayashizaki

**Affiliations:** The Jackson Laboratory, United States of America

## Abstract

For several decades, only a limited number of noncoding RNAs, such as ribosomal and transfer RNA, have been studied in any depth. In recent years, additional species of noncoding RNAs have increasingly been discovered. Of these, small RNA species attract particular interest because of their essential roles in processes such as RNA silencing and modifications. Detailed analyses revealed several pathways associated with the function of small RNAs. Although these pathways show evolutional conservation, there are substantial differences. Advanced technologies to profile RNAs have accelerated the field further resulting in the discovery of an increasing number of novel species, suggesting that we are only just beginning to appreciate the complexity of small RNAs and their functions. Here, we review recent progress in novel small RNA exploration, including discovered small RNA species, their pathways, and devised technologies.

## Introduction

There is substantial interest in noncoding RNAs (ncRNAs), which play an essential role in complex biological systems without encoding for proteins. Only a limited number of ncRNAs, such as ribosomal RNA (rRNA) and transfer RNA (tRNA), have previously been characterized in any depth. Recent studies revealed many novel ncRNAs, covering a wide range of sizes [1]. RNA molecules have several functions including catalytic activity and ability to act as a structural component. Of these functions, the ability to specify a nucleic acid sequence is superior compared to proteins. A common way in which ncRNA contributes to biological processes is through the ribonucleoprotein (RNP) complex, where its role is to guide recognition of nucleic acid target sequences relying upon sequence complementarity [2]. Small RNA molecules are widely utilized in this type of machinery, and are involved in important biological processes [3]. Exploration of novel small RNA species and their functions attracts substantial interest. The advent of recent technologies to profile cellular RNAs, such as high-throughput sequencing and microarray, coupled with computational analysis, has contributed to rapid progress in this field. Here, we review the recently discovered small RNA species and their pathways in a view of conservations and differences between higher eukaryotes. We also summarize recent exploration efforts of novel small RNAs based on devised technologies to provide a perspective for the future.

## RNA Silencing–Related Small RNAs

RNA silencing of endogenous genes, viruses, and selfish genomic elements is a regulatory process that relies on small RNA molecules, approximately 22 nucleotides long [4]. The trigger of RNA silencing is an RNA molecule harboring a duplex. Such a molecule is processed with the following steps: (i) small RNA production: a precursor RNA is cleaved to produce a small double-stranded RNA (dsRNA), where the precursor can be a hairpin-structured RNA or a long dsRNA in the case of microRNA (miRNA) or small interfering RNA (siRNA), respectively; (ii) RNP assembly: the resulting small RNA is loaded into an RNP complex, and (iii) gene silencing: the RNP suppresses its target gene, where the target recognition is guided by the loaded small RNA, and the silencing activity is mediated by the proteins composing the RNP at the post-transcriptional or transcriptional level [5].

**Figure 1:**
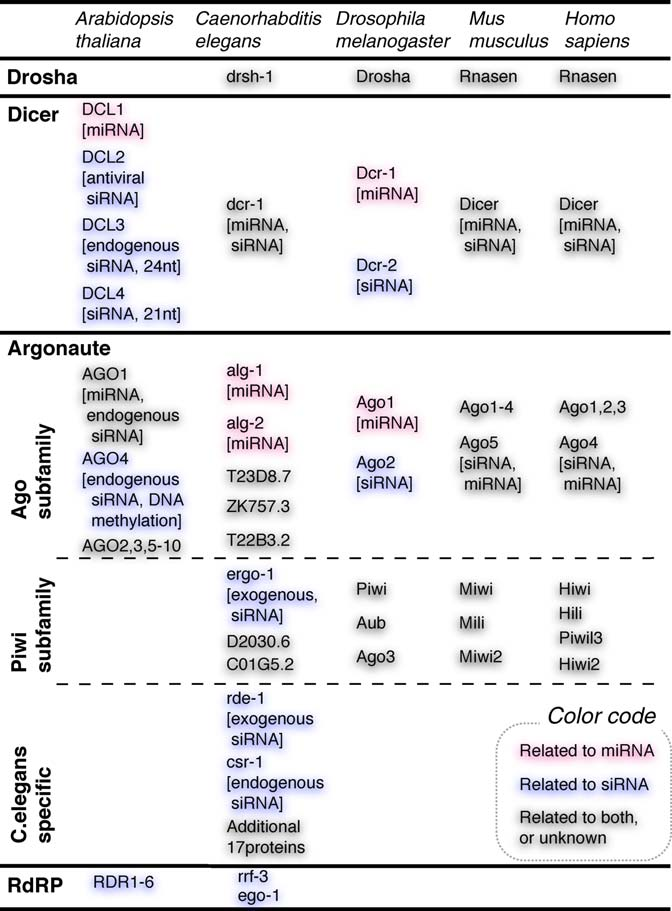
RNA Silencing Related Proteins and Their Related Pathways RNA silencing–related proteins are categorized by their families and subfamilies. Their pathways are indicated in parentheses.

This machinery is adopted in a wide range of organisms. Although the overall pathways resemble each other, there are substantial differences between organisms (Figure 1). In metazoans, two RNase III endonucleases, Drosha and Dicer, contribute to a process of small RNA production. Drosha cleaves a long primary transcript including a stem-loop (termed primary miRNA, or pri-miRNA) near the base of the stem to release a hairpin structure, termed precursor miRNA (pre-miRNA). Dicer cleaves pre-miRNA or a long dsRNA (precursor of siRNA) to produce a small dsRNA, only one strand of which is loaded into the RNP [6]. Homo sapiens, Mus musculus, and Caenorhabditis elegans have only one Dicer gene, which contributes to both miRNA and siRNA production. On the other hand, these two roles are encoded by distinct genes in several organisms. Drosophila melanogaster has two proteins of Dicer, DCR-1, and DCR-2, which are used for miRNA and siRNA, respectively [7]. Arabidopsis thaliana has four Dicer orthologues, DCL1 to DCL4, but no Drosha. DCL1 contributes to miRNA production, DCL2 to 22nt siRNA from invading viruses, and DCL3 to 24nt siRNA from endogenous genes [8,9].

There are also differences in the effector complexes participating in RNA silencing, siRNP, or miRNP. Their main component is Argonaute, which has two principal domains: an RNA-binding PAZ domain at the N-terminus and RNase-like Piwi domain at the C-terminus. The Argonaute protein family consists of the Ago subfamily, the Piwi subfamily, and the C. elegans specific subfamily (Figure 1) [10]. Mammalian AGO subfamily members contribute to both the siRNA and miRNA pathways. In contrast, AGO1 contributes only to the miRNA pathway, and AGO2 only to the siRNA pathway in D. melanogaster [7]. The many members in C. elegans have also been suggested to have a distinction in their roles [11]. Only ALG contributes to miRNA processing, whereas RDE-1 and ERGO-1 cleave exogenous and endogenous dsRNA to produce siRNA. Intriguingly, the Piwi subfamily is not found in A. thaliana. This organism has only AGO subfamily members, where AGO1 is involved in miRNA and endogenous siRNA, and AGO4 in DNA methylation through endogenous siRNA [9].

There is an additional organism-dependent pathway: an amplification of small RNAs relying upon RNA-dependent RNA polymerase (RdRP), which currently has been found only in C. elegans and A. thaliana. The polymerase synthesizes dsRNAs from RNAs cleaved by siRNP or miRNP, and the synthesized dsRNAs are used as siRNA precursors [12]. This process contributes to the amplification of siRNA and the subsequent silencing effect. In plants, two cleavage events often trigger siRNA biogenesis by this amplification pathway [13]. RdRP activity has also been observed in D. melanogaster, although its homolog has not yet been identified [14].

### Endogenous siRNA variations.

siRNAs were originally found as processed products of introduced long dsRNA, but subsequent analysis has revealed endogenous siRNAs with several origins. Repeat-associated siRNA (rasiRNA) originate from repetitive sequences, such as transposable elements, that have the ability to replicate themselves independent of their host organisms. rasiRNAs are found in both the sense and antisense strands of the transposable elements, and there are predominant biases in the strand from which the RNAs are derived [15–20]. rasiRNAs are suggested to repress the transposable elements themselves or mRNAs harboring sequence complementary to them. Besides post-transcriptional level silencing, rasiRNAs have been shown to be involved in transcriptional level silencing in plants through chromatin modifications [15,16]. Repetitive sequences are frequently found in chromatin domains, and it is suggested that rasiRNAs contribute to the regulation of chromatin status.

Trans-acting siRNA (ta-siRNA) is a class of siRNA that targets other genes rather than the gene producing the siRNA itself [21]. It is found only in plants, and surprisingly it is derived from an mRNA cleaved by a miRNA. A cleaved product of the mRNA attacked by a miRNP is used as a template to synthesize double-strand RNA with RdRP, and the synthesized product triggers RNA silencing to repress other genes [22,23].

### Putatively siRNA-related RNAs.

Novel types of ncRNAs that resemble siRNAs have also been discovered, although their function still remains unclear. Piwi-interacting RNAs (piRNAs) are potentially a third class of small RNAs involved in RNA silencing [24–26]. Piwi-related proteins compose a subfamily belonging to the AGO family as described above, and they are specifically expressed in the germline. In fact, Piwi of D. melanogaster and Miwi2 of mouse are essential for germline stem cells, which suggests a common machinery between these organisms [27]. In fact, piRNAs are commonly 25nt–29nt long, which are slightly longer than miRNAs and siRNAs, and they are clustered into a limited number of loci on the genome, where only one strand encodes piRNA mainly. Recent analysis suggests a model of its biogenesis in fly: piRNA attacks a transcribed transposon, and the cleaved transposon contributes to piRNA production [28]. Surprisingly, the three members of the piwi subfamily, Piwi, Aub, and Ago3, play distinct roles in the model: Piwi and Aub bind to piRNA specifically, which is the antisense of the transposon, while Ago3 binds the cleaved fragment of the targeted transposon. The interplay between piRNAs that are sense and antisense to transposable elements is suggested to be conserved in mouse [29]. However, any long transcripts derived from the piRNA loci, potential precursors of piRNA, have not yet been found. And mouse piRNAs are not significantly related to repeats, while a substantial part of fly piRNAs are derived from repeats [24,25]. The piRNA pathway including its biogenesis still remains to be studied in detail.

A class of 21nt-long RNAs that is distinct from miRNA and siRNA has been found in C. elegans. These RNAs, termed 21U-RNA [30], are similar to piRNA in a few aspects: an uridine is frequently found at their 5′ end, and their origins are clustered on the genome. However, their clusters span large regions (2–3 Mbp), compared to the piRNA clusters (approximately 100 kbp). The 21U-RNAs also share two motifs in their upstream (less than 50 bp), whereas no motifs have so far been found in biogenesis of small RNA involving RNA silencing [30]. C. elegans specific subfamily piwi (Figure 1) is expected to comprise species-specific pathways, and some of them might be related to the 21U-RNAs.

## snoRNA/scaRNA

Small nucleolar RNA (snoRNA), another class of small RNA discovered recently, contributes to RNA modification of ribosomal RNAs (rRNAs), small nuclear RNAs (snRNAs), and, putatively, other RNAs [31]. Two families of snoRNA have been revealed to catalyze distinct modifications: the C/D box family, for 2′-O-methylation, and the H/ACA box, for pseudouridylation. The former possesses two motifs at the 5′- and 3′-ends (termed C and D boxes), imperfect copies of the motifs (C′ and D′ boxes), and guide sequences to specify target RNAs. C/D box snoRNA forms an RNP complex with four proteins: FBL (fibrillarin, a methyltransferase), NOL5A (Nop56), NOP5/NOP58, and NHP2L1 (Snu13). The H/ACA box snoRNA possesses two hairpins, which contain internal loops to form pseudo-knot structures with its target RNA, and two single-stranded regions containing two motifs (H and ACA boxes). This also comprises RNP with distinct four proteins: DKC1 (dyskerin, a pseudouridine synthase), NOLA1 (Gar1p), NOLA2 (Nhp2p), and NOLA3 (Nop10p) [32]. These snoRNPs mediate the modifications of the targeted RNA, where their target sites are recognized by complementary (guide) sequences within the snoRNAs. Orphan snoRNAs, whose guide sequences are not complementary to rRNA or snRNA, have also been observed [33]. Notably, one of these orphan snoRNAs, HBII-52, which is located in an imprinted locus, has been revealed to regulate alternative splicing of the serotonin receptor 2C by relying upon sequence complementarity. Loss of this snoRNA produces different isoforms of mRNA, which are likely to cause the Prader-Willi syndrome [34]. This finding suggests that the other orphan snoRNAs are potentially involved in splicing machinery as well as in RNA modification. Intriguingly, composite RNAs harboring both C/D boxes and H/ACA boxes have been found. They are localized to Cajal bodies, conserved subnuclear organelles in the nucleoplasm, and are termed scaRNA. They are suggested to mediate both of the two modifications, 2′-O-methylation and pseudouridylation, relying upon the corresponding motifs [35,36].

## Small RNA Exploration

Technologies to profile cellular RNA, sometimes termed RNomics [37,38], have led to recent discoveries in the field of small RNA. Their advances drastically extend the range that can be explored. The approaches to profile cellular RNAs are mainly classified into four categories [38]: (i) RNA direct sequencing, (ii) cDNA cloning followed by sequencing, (iii) hybridization-based detection, and (iv) genomic SELEX. The first category, RNA direct sequencing, is a classical method applicable for a very limited number of RNAs, which are highly abundant and distinguishable from other species relying upon just length, like tRNA and rRNA. The fourth category, genomic SELEX, identifies possible RNA sequences for binding a specific protein through in vitro synthesis of RNAs based on the genome. Although it has a benefit in its independence from samples expressing target RNAs, its application is quite limited. The remaining two, cDNA sequencing and the hybridization-based approach, are the most commonly used methods for recent exploration efforts.

### cDNA sequencing.

**Figure 2:**
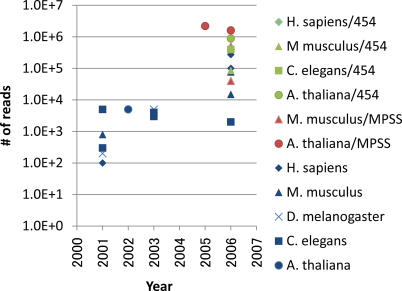
Large-Scale Sequencing of Small RNAs The number of sequence reads within the recent studies of small RNA exploration with large-scale sequencing. Targeted organisms and adopted sequencing technologies are distinguished by colored markers. The original data is provided as Table S1.

A widely used approach to explore small RNAs is random sequencing of size-fractionated RNAs, which requires linker ligation to cellular RNAs, reverse-transcription, PCR amplification, concatemerization, cloning, and sequencing [39]. Based on its ability to explore unpredicted RNAs, this approach initiated the systematic exploration of novel miRNAs [40–42], and subsequently it has been applied to profile additional RNA species in a wide range of samples, such as RNAs extracted from various organisms, and mutants, and RNAs immunoprecipitated with a related protein [13,24,25,28,30,43–45]. A benefit of random sequencing is its ability to extract information about the abundance of different RNA species included in the sample. As the likelihood of a molecule being sequenced correlates with its abundance, rare species have a small chance of being discovered in small-scale sequencing. This limitation is being addressed by the development of highly parallel sequencing technologies, such as MPSS [46] and 454 pyrosequencing [47]. These large-scale sequencing techniques enable the reading of small RNAs several hundred thousand times (Figure 2, Table S1), which make it possible to detect rare species and to quantify RNA abundances with better accuracy. The number of reads obtained through random sequencing of cDNA will not necessarily reflect the original proportion, due to different efficiencies in reverse transcription of RNA into cDNA depending on secondary structure and/or modifications [38]. However, it is still possible to compare abundances of detected RNAs across samples because reverse-transcription efficiency is expected to depend on the RNA itself, not on which samples were used.

### Hybridization-based detection.

Hybridization-based detection systems, such as northern blots and microarrays, are used to detect and/or quantify expression of small RNAs [37,38] as well as mRNAs. Northern blots are commonly used to confirm small RNAs detected with other methods and have also been devised for expression profiling of more than 100 miRNAs [48]. Its sensitivity is comparatively limited, but the limitation can be addressed by the use of locked nucleic acids (LNA) as probes [49] and the use of the soluble carbodiimide to cross-link RNA to nylon membranes [50]. Microarrays are utilized for large-scale profiling of small RNAs [51,52], and their sensitivities and specificities can also be improved with the use of beads [53], LNA [54], and incorporation of hairpin structures to probes [55]. In the context of novel miRNA exploration, customized microarrays are used in combination with computational prediction [43,56]. Such predictions are not necessarily optimal, and will predict slightly different sequences in several cases. In order to detect true miRNAs from such predictions, RNA-primed array-based Klenow extension (RAKE) has been used to clearly distinguish distinct borders of a mature miRNA with tiling probes covering the regions proximal to the predictions [43]. Another study performed sequence-directed cDNA cloning and sequencing following a microarray analysis to confirm and determine the detected sequences [56]. Besides miRNA analysis, siRNAs and rasiRNAs have been analyzed with microarrays, in particular with high-density (tiling) microarrays. In the study of siRNAs derived from scattered sense and antisense regions within a locus, the use of tiling arrays to profile the entire region revealed distinct features between siRNAs and rasiRNAs [19]. In another study, whole genome tiling array is used to profile small RNAs, as well as long RNAs derived from the nucleus and the cytosol. This enabled the profiling of the production process of small RNAs on a whole-genome scale [57].

### Computational analysis.

The experimental approaches described above require a coupling to computational analysis, such as the prediction of small RNAs and characterization of discovered RNAs. The sequencing approach requires classification of obtained RNAs in order to get a complete picture of the RNA composition in the analyzed samples, and to select RNAs of interest in subsequent analyses. A major approach in the classification of small RNAs is mapping them onto the genome with subsequent comparison to genome annotations. Databases collecting ncRNA sequences (and profiles in Rfam [58]) of specific interest (Table 1) are also available for such classifications.

**Table 1:**
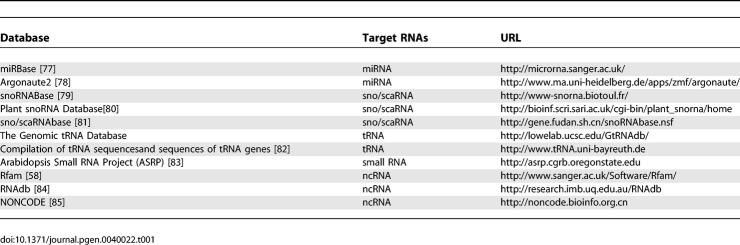
ncRNA Databases

Except in the case of the whole-genome tiling arrays, the hybridization-based approach requires target RNA sequences to design probes in advance of experiments. Computational prediction of miRNA has been performed in many studies, which mainly rely upon the secondary structure of the miRNA precursor [59,60], thermodynamic stability [61], and/or genome conservation between species [62–64]. Several studies have succeeded in validating the predicted RNAs experimentally and demonstrating the accuracy of the prediction methods [65]. Prediction of snoRNA has also been performed computationally, relying upon features such as secondary structure, antisense sequence to putative target, and the presence of C/D and H/ACA boxes [66–70]. Recent analyses have revealed unexpected RNAs even within these classes, such as species-specific (not conserved among species) miRNA and orphan snoRNA with unknown targets, as described above. In particular, genome conservation is used broadly to find functional elements within the genome, but this does not necessarily mean that nonconserved regions have no function. Improvements of computational methods with consideration of the recent findings are used to explore further novel RNAs [56,69,71].

## Discussion

Recent post-genome analyses have revealed that a large fraction of the genome, more than 60%–70%, can be transcribed [72,73]. Considering that small RNAs are derived from intergenic regions, introns, exons, and repetitive sequences originally thought to be unimportant or junk, all transcripts are potential sources of functional small RNAs. Although a limited fraction of the cellular RNAs have been characterized functionally [1], it is expected that a substantial number of the small RNA species and their related pathways still remain unrevealed. Recent studies have demonstrated that synthetic RNA duplexes harboring sequences complementary to promoters rather than mRNA can contribute to gene activation and repression [74–76]. No endogenous RNAs have previously been found to have such a function. This does not necessarily mean that no endogenous RNAs are involved in this machinery, but rather suggests the possibility that there are still unrevealed small RNA pathways.

An approach to tackle a hidden species is a systematic and exhaustive exploration of various samples using the enhanced technologies. As seen in Figure 1, proteins related to small RNA pathways do not necessarily play the same roles in different organisms. RNA profiling with perturbation and/or immunoprecipitation of each related protein in each organism may lead to discoveries of novel species. Another target would be RNA species specifically localized in subcellular components. In-depth profiling of nuclear organelles, such as the nucleolus, Cajal bodies, and P-bodies, has not yet been completed. This would contribute to the revelation of novel RNA species, as well as the trafficking and processing pathways of RNA molecules.

## Supporting Information

Table S1 Large-Scale Sequencing of Small RNAs The original data of Figure 2 including the number of sequence reads and their references.
(38 KB XLS)

## References

[pgen-0040022-b001] Mattick JS, Makunin IV (2006). Non-coding RNA. Hum Mol Genet.

[pgen-0040022-b002] Huttenhofer A, Schattner P (2006). The principles of guiding by RNA: chimeric RNA-protein enzymes. Nat Rev Genet.

[pgen-0040022-b003] Mattick JS, Makunin IV (2005). Small regulatory RNAs in mammals. Hum Mol Genet.

[pgen-0040022-b004] Kim VN (2005). Small RNAs: classification, biogenesis, and function. Mol Cells.

[pgen-0040022-b005] Almeida R, Allshire RC (2005). RNA silencing and genome regulation. Trends Cell Biol.

[pgen-0040022-b006] Bartel DP (2004). MicroRNAs: genomics, biogenesis, mechanism, and function. Cell.

[pgen-0040022-b007] Kavi HH, Fernandez HR, Xie W, Birchler JA (2005). RNA silencing in Drosophila. FEBS Lett.

[pgen-0040022-b008] Bonnet E, Van de Peer Y, Rouze P (2006). The small RNA world of plants. New Phytol.

[pgen-0040022-b009] Herr AJ (2005). Pathways through the small RNA world of plants. FEBS Lett.

[pgen-0040022-b010] Parker JS, Barford D (2006). Argonaute: A scaffold for the function of short regulatory RNAs. Trends Biochem Sci.

[pgen-0040022-b011] Yigit E, Batista PJ, Bei Y, Pang KM, Chen CC (2006). Analysis of the C. elegans Argonaute family reveals that distinct Argonautes act sequentially during RNAi. Cell.

[pgen-0040022-b012] Nishikura K (2001). A short primer on RNAi: RNA-directed RNA polymerase acts as a key catalyst. Cell.

[pgen-0040022-b013] Axtell MJ, Jan C, Rajagopalan R, Bartel DP (2006). A two-hit trigger for siRNA biogenesis in plants. Cell.

[pgen-0040022-b014] Lipardi C, Wei Q, Paterson BM (2001). RNAi as random degradative PCR: siRNA primers convert mRNA into dsRNAs that are degraded to generate new siRNAs. Cell.

[pgen-0040022-b015] Lippman Z, Gendrel AV, Black M, Vaughn MW, Dedhia N (2004). Role of transposable elements in heterochromatin and epigenetic control. Nature.

[pgen-0040022-b016] Liu J, He Y, Amasino R, Chen X (2004). siRNAs targeting an intronic transposon in the regulation of natural flowering behavior in Arabidopsis. Genes Dev.

[pgen-0040022-b017] Sijen T, Plasterk RH (2003). Transposon silencing in the Caenorhabditis elegans germ line by natural RNAi. Nature.

[pgen-0040022-b018] Aravin AA, Lagos-Quintana M, Yalcin A, Zavolan M, Marks D (2003). The small RNA profile during Drosophila melanogaster development. Dev Cell.

[pgen-0040022-b019] Vagin VV, Sigova A, Li C, Seitz H, Gvozdev V (2006). A distinct small RNA pathway silences selfish genetic elements in the germline. Science.

[pgen-0040022-b020] Watanabe T, Takeda A, Tsukiyama T, Mise K, Okuno T (2006). Identification and characterization of two novel classes of small RNAs in the mouse germline: retrotransposon-derived siRNAs in oocytes and germline small RNAs in testes. Genes Dev.

[pgen-0040022-b021] Vazquez F, Vaucheret H, Rajagopalan R, Lepers C, Gasciolli V (2004). Endogenous trans-acting siRNAs regulate the accumulation of Arabidopsis mRNAs. Mol Cell.

[pgen-0040022-b022] Allen E, Xie Z, Gustafson AM, Carrington JC (2005). microRNA-directed phasing during trans-acting siRNA biogenesis in plants. Cell.

[pgen-0040022-b023] Yoshikawa M, Peragine A, Park MY, Poethig RS (2005). A pathway for the biogenesis of trans-acting siRNAs in Arabidopsis. Genes Dev.

[pgen-0040022-b024] Aravin A, Gaidatzis D, Pfeffer S, Lagos-Quintana M, Landgraf P (2006). A novel class of small RNAs bind to MILI protein in mouse testes. Nature.

[pgen-0040022-b025] Girard A, Sachidanandam R, Hannon GJ, Carmell MA (2006). A germline-specific class of small RNAs binds mammalian Piwi proteins. Nature.

[pgen-0040022-b026] Lau NC, Seto AG, Kim J, Kuramochi-Miyagawa S, Nakano T (2006). Characterization of the piRNA complex from rat testes. Science.

[pgen-0040022-b027] Carmell MA, Girard A, van de Kant HJ, Bourc'his D, Bestor TH (2007). MIWI2 is essential for spermatogenesis and repression of transposons in the mouse male germline. Dev Cell.

[pgen-0040022-b028] Brennecke J, Aravin AA, Stark A, Dus M, Kellis M (2007). Discrete small RNA-generating loci as master regulators of transposon activity in Drosophila. Cell.

[pgen-0040022-b029] Aravin AA, Sachidanandam R, Girard A, Fejes-Toth K, Hannon GJ (2007). Developmentally regulated piRNA clusters implicate MILI in transposon control. Science.

[pgen-0040022-b030] Ruby JG, Jan C, Player C, Axtell MJ, Lee W (2006). Large-scale sequencing reveals 21U-RNAs and additional microRNAs and endogenous siRNAs in C. elegans. Cell.

[pgen-0040022-b031] Kiss T (2001). Small nucleolar RNA-guided post-transcriptional modification of cellular RNAs. EMBO J.

[pgen-0040022-b032] Kiss T (2004). Biogenesis of small nuclear RNPs. J Cell Sci.

[pgen-0040022-b033] Huttenhofer A, Kiefmann M, Meier-Ewert S, O'Brien J, Lehrach H (2001). RNomics: an experimental approach that identifies 201 candidates for novel, small, non-messenger RNAs in mouse. EMBO J.

[pgen-0040022-b034] Kishore S, Stamm S (2006). The snoRNA HBII-52 regulates alternative splicing of the serotonin receptor 2C. Science.

[pgen-0040022-b035] Jady BE, Kiss T (2001). A small nucleolar guide RNA functions both in 2′-O-ribose methylation and pseudouridylation of the U5 spliceosomal RNA. EMBO J.

[pgen-0040022-b036] Darzacq X, Jady BE, Verheggen C, Kiss AM, Bertrand E (2002). Cajal body-specific small nuclear RNAs: a novel class of 2′-O-methylation and pseudouridylation guide RNAs. EMBO J.

[pgen-0040022-b037] Aravin A, Tuschl T (2005). Identification and characterization of small RNAs involved in RNA silencing. FEBS Lett.

[pgen-0040022-b038] Huttenhofer A, Vogel J (2006). Experimental approaches to identify non-coding RNAs. Nucleic Acids Res.

[pgen-0040022-b039] Ambros V, Lee RC (2004). Identification of microRNAs and other tiny noncoding RNAs by cDNA cloning. Methods Mol Biol.

[pgen-0040022-b040] Lagos-Quintana M, Rauhut R, Lendeckel W, Tuschl T (2001). Identification of novel genes coding for small expressed RNAs. Science.

[pgen-0040022-b041] Lau NC, Lim LP, Weinstein EG, Bartel DP (2001). An abundant class of tiny RNAs with probable regulatory roles in Caenorhabditis elegans. Science.

[pgen-0040022-b042] Lee RC, Ambros V (2001). An extensive class of small RNAs in Caenorhabditis elegans. Science.

[pgen-0040022-b043] Berezikov E, van Tetering G, Verheul M, van de Belt J, van Laake L (2006). Many novel mammalian microRNA candidates identified by extensive cloning and RAKE analysis. Genome Res.

[pgen-0040022-b044] Berezikov E, Thuemmler F, van Laake LW, Kondova I, Bontrop R (2006). Diversity of microRNAs in human and chimpanzee brain. Nat Genet.

[pgen-0040022-b045] Lu C, Kulkarni K, Souret FF, MuthuValliappan R, Tej SS (2006). MicroRNAs and other small RNAs enriched in the Arabidopsis RNA-dependent RNA polymerase-2 mutant. Genome Res.

[pgen-0040022-b046] Brenner S, Johnson M, Bridgham J, Golda G, Lloyd DH (2000). Gene expression analysis by massively parallel signature sequencing (MPSS) on microbead arrays. Nat Biotechnol.

[pgen-0040022-b047] Margulies M, Egholm M, Altman WE, Attiya S, Bader JS (2005). Genome sequencing in microfabricated high-density picolitre reactors. Nature.

[pgen-0040022-b048] Sempere LF, Freemantle S, Pitha-Rowe I, Moss E, Dmitrovsky E (2004). Expression profiling of mammalian microRNAs uncovers a subset of brain-expressed microRNAs with possible roles in murine and human neuronal differentiation. Genome Biol.

[pgen-0040022-b049] Valoczi A, Hornyik C, Varga N, Burgyan J, Kauppinen S (2004). Sensitive and specific detection of microRNAs by northern blot analysis using LNA-modified oligonucleotide probes. Nucleic Acids Res.

[pgen-0040022-b050] Pall GS, Codony-Servat C, Byrne J, Ritchie L, Hamilton A (2007). Carbodiimide-mediated cross-linking of RNA to nylon membranes improves the detection of siRNA, miRNA and piRNA by northern blot. Nucleic Acids Res.

[pgen-0040022-b051] Barad O, Meiri E, Avniel A, Aharonov R, Barzilai A (2004). MicroRNA expression detected by oligonucleotide microarrays: system establishment and expression profiling in human tissues. Genome Res.

[pgen-0040022-b052] Liu CG, Calin GA, Meloon B, Gamliel N, Sevignani C (2004). An oligonucleotide microchip for genome-wide microRNA profiling in human and mouse tissues. Proc Natl Acad Sci U S A.

[pgen-0040022-b053] Lu J, Getz G, Miska EA, Alvarez-Saavedra E, Lamb J (2005). MicroRNA expression profiles classify human cancers. Nature.

[pgen-0040022-b054] Castoldi M, Schmidt S, Benes V, Noerholm M, Kulozik AE (2006). A sensitive array for microRNA expression profiling (miChip) based on locked nucleic acids (LNA). Rna.

[pgen-0040022-b055] Wang H, Ach RA, Curry B (2007). Direct and sensitive miRNA profiling from low-input total RNA. Rna.

[pgen-0040022-b056] Bentwich I, Avniel A, Karov Y, Aharonov R, Gilad S (2005). Identification of hundreds of conserved and nonconserved human microRNAs. Nat Genet.

[pgen-0040022-b057] Kapranov P, Cheng J, Dike S, Nix DA, Duttagupta R (2007). RNA maps reveal new RNA classes and a possible function for pervasive transcription. Science.

[pgen-0040022-b058] Griffiths-Jones S, Moxon S, Marshall M, Khanna A, Eddy SR (2005). Rfam: annotating non-coding RNAs in complete genomes. Nucleic Acids Res.

[pgen-0040022-b059] Zuker M (1989). On finding all suboptimal foldings of an RNA molecule. Science.

[pgen-0040022-b060] Hofacker IL, Fontana W, Stadler PF, Bonhoeffer LS, Tacker M (1994). Fast folding and comparison of RNA secondary structures. Monatsh Chem.

[pgen-0040022-b061] Washietl S, Hofacker IL, Stadler PF (2005). Fast and reliable prediction of noncoding RNAs. Proc Natl Acad Sci U S A.

[pgen-0040022-b062] Lim LP, Glasner ME, Yekta S, Burge CB, Bartel DP (2003). Vertebrate microRNA genes. Science.

[pgen-0040022-b063] Berezikov E, Guryev V, van de Belt J, Wienholds E, Plasterk RH (2005). Phylogenetic shadowing and computational identification of human microRNA genes. Cell.

[pgen-0040022-b064] Washietl S, Hofacker IL, Lukasser M, Huttenhofer A, Stadler PF (2005). Mapping of conserved RNA secondary structures predicts thousands of functional noncoding RNAs in the human genome. Nat Biotechnol.

[pgen-0040022-b065] Bentwich I (2005). Prediction and validation of microRNAs and their targets. FEBS Lett.

[pgen-0040022-b066] Barneche F, Gaspin C, Guyot R, Echeverria M (2001). Identification of 66 box C/D snoRNAs in Arabidopsis thaliana: extensive gene duplications generated multiple isoforms predicting new ribosomal RNA 2′-O-methylation sites. J Mol Biol.

[pgen-0040022-b067] Lowe TM, Eddy SR (1999). A computational screen for methylation guide snoRNAs in yeast. Science.

[pgen-0040022-b068] Schattner P, Decatur WA, Davis CA, Ares M, Fournier MJ (2004). Genome-wide searching for pseudouridylation guide snoRNAs: analysis of the Saccharomyces cerevisiae genome. Nucleic Acids Res.

[pgen-0040022-b069] Yang JH, Zhang XC, Huang ZP, Zhou H, Huang MB (2006). snoSeeker: an advanced computational package for screening of guide and orphan snoRNA genes in the human genome. Nucleic Acids Res.

[pgen-0040022-b070] Fedorov A, Stombaugh J, Harr MW, Yu S, Nasalean L (2005). Computer identification of snoRNA genes using a Mammalian Orthologous Intron Database. Nucleic Acids Res.

[pgen-0040022-b071] Rigoutsos I, Huynh T, Miranda K, Tsirigos A, McHardy A (2006). Short blocks from the noncoding parts of the human genome have instances within nearly all known genes and relate to biological processes. Proc Natl Acad Sci U S A.

[pgen-0040022-b072] Carninci P, Kasukawa T, Katayama S, Gough J, Frith MC (2005). The transcriptional landscape of the mammalian genome. Science.

[pgen-0040022-b073] ENCODE Project Consortium (2007). Identification and analysis of functional elements in 1% of the human genome by the ENCODE pilot project. Nature.

[pgen-0040022-b074] Janowski BA, Huffman KE, Schwartz JC, Ram R, Hardy D (2005). Inhibiting gene expression at transcription start sites in chromosomal DNA with antigene RNAs. Nat Chem Biol.

[pgen-0040022-b075] Janowski BA, Huffman KE, Schwartz JC, Ram R, Nordsell R (2006). Involvement of AGO1 and AGO2 in mammalian transcriptional silencing. Nat Struct Mol Biol.

[pgen-0040022-b076] Janowski BA, Younger ST, Hardy DB, Ram R, Huffman KE (2007). Activating gene expression in mammalian cells with promoter-targeted duplex RNAs. Nat Chem Biol.

[pgen-0040022-b077] Griffiths-Jones S, Grocock RJ, van Dongen S, Bateman A, Enright AJ (2006). miRBase: microRNA sequences, targets and gene nomenclature. Nucleic Acids Res.

[pgen-0040022-b078] Shahi P, Loukianiouk S, Bohne-Lang A, Kenzelmann M, Kuffer S (2006). Argonaute—a database for gene regulation by mammalian microRNAs. Nucleic Acids Res.

[pgen-0040022-b079] Lestrade L, Weber MJ (2006). snoRNA-LBME-db, a comprehensive database of human H/ACA and C/D box snoRNAs. Nucleic Acids Res.

[pgen-0040022-b080] Brown JW, Echeverria M, Qu LH, Lowe TM, Bachellerie JP (2003). Plant snoRNA database. Nucleic Acids Res.

[pgen-0040022-b081] Xie J, Zhang M, Zhou T, Hua X, Tang L (2007). Sno/scaRNAbase: a curated database for small nucleolar RNAs and cajal body-specific RNAs. Nucleic Acids Res.

[pgen-0040022-b082] Sprinzl M, Vassilenko KS (2005). Compilation of tRNA sequences and sequences of tRNA genes. Nucleic Acids Res.

[pgen-0040022-b083] Gustafson AM, Allen E, Givan S, Smith D, Carrington JC (2005). ASRP: the Arabidopsis Small RNA Project Database. Nucleic Acids Res.

[pgen-0040022-b084] Pang KC, Stephen S, Dinger ME, Engstrom PG, Lenhard B (2007). RNAdb 2.0—an expanded database of mammalian non-coding RNAs. Nucleic Acids Res.

[pgen-0040022-b085] Liu C, Bai B, Skogerbo G, Cai L, Deng W (2005). NONCODE: an integrated knowledge database of non-coding RNAs. Nucleic Acids Res.

